# Signs and symptoms of internalizing and externalizing disorders and opportunities for clinical translation

**DOI:** 10.1038/s44277-025-00031-2

**Published:** 2025-06-05

**Authors:** Adrienne L. Romer

**Affiliations:** 1 Department of Psychology, Virginia Polytechnic Institute and State University, Blacksburg, VA USA; 2 Center for Depression, Anxiety and Stress Research, McLean Hospital, Belmont, MA USA

**Keywords:** Human behaviour, Psychiatric disorders, Diseases of the nervous system

**Fig. 1:**
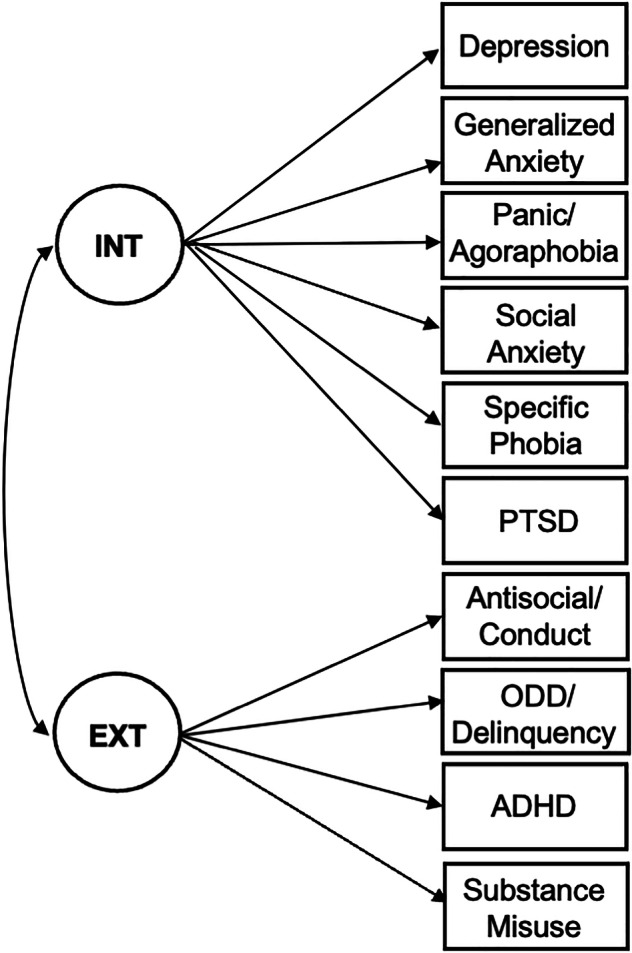
Factor-analytic model of internalizing and externalizing dimensions. Empirical studies using factor analysis consistently have identified this latent two-factor structure of psychopathology in a wide range of study samples across the lifespan. INT Internalizing, EXT externalizing; circles = latent factors; rectangles = measured symptoms, single-headed arrows =factor loadings, double-headed arrows = correlation, PSTD posttraumatic stress disorder, ODD oppositional defiant disorder, ADHD attention-deficit/hyperactivity disorder.

Comorbidity, dimensionality, and heterogeneity of psychiatric symptoms have been major challenges for the field of psychiatry. Difficulties identifying causes, biomarkers, and treatments with specificity to individual disorders has indicated a need to shift away from the study of discrete psychiatric disorders towards examining transdiagnostic (i.e., cross-cutting) dimensional (i.e., on a continuum) features shared across categorical disorders. In support of this shift, the NIMH Research Domain Criteria [1] and Hierarchical Taxonomy of Psychopathology (HiTOP) [2] classification systems were developed based on empirical studies demonstrating that groups of disorders and symptoms predictably co-occur as part of a smaller number of transdiagnostic dimensions. For example, depression and anxiety emerge in the same individuals and comprise an Internalizing dimension and antisocial behavior and substance misuse emerge in the same individuals and comprise an Externalizing dimension. These dimensions were termed “Internalizing” and “Externalizing” because of the way their core symptoms are typically expressed, with anxiety and depressive symptoms experienced more inwardly than the antisocial and substance-use symptoms, which are typically expressed outwardly. Internalizing and Externalizing dimensions are identified using factor-analytic methods that capture the covariation and severity of psychiatric disorders (Fig. 1).

These Internalizing and Externalizing dimensions have been identified in many samples across the lifespan, are robust to differences in culture, gender, ethnicity, sexual orientation, and assessment methodologies, and have distinct genetic and neural vulnerabilities shared across the disorders within each dimension [3]. Further, the dimensions reliably predict disorder onset, course, and treatment response better than individual disorder categories [3], indicating their validity, reliability, and clinical utility over and above categorical disorders. Thus, this Internalizing-Externalizing framework can improve our understanding of the nature and treatment of psychiatric disorders across traditional diagnostic boundaries. Table 1 shows a glossary of the core signs and symptoms, psychiatric disorders, developmental progression, etiology, and example questionnaire items of the Internalizing and Externalizing dimensions.

**Table 1:** Glossary of signs, symptoms, disorders, example questionnaire items, development, and etiological markers of internalizing and externalizing disorders.

	Internalizing	Externalizing
Definition	Inward or internal expression of psychiatric symptoms	Outward or external expression of psychiatric symptoms
Core Signs and Symptoms	Anxiousness, emotional lability, perseveration, submissiveness, dysphoria, agitation, anhedonia, suicidality, anxiety, panic, avoidance, hyperarousal, irritability	Impulsivity, irresponsibility, distractibility, risk taking, substance use, aggression, impatient urgency, excitement seeking, attention seeking, callousness, deceitfulness, grandiosity, manipulativeness, dominance
Categorical Disorders	Major Depressive Disorder, Generalized Anxiety Disorder, Persistent Depressive Disorder (Dysthymia), Posttraumatic Stress Disorder, Panic Disorder, Agoraphobia, Social Anxiety Disorder, Specific Phobias, and Separation Anxiety Disorder	Conduct Disorder, Oppositional Defiant Disorder, Attention/Deficit Hyperactivity Disorder, Antisocial Personality Disorder, and Alcohol, Nicotine, and other Substance-Related Disorders
Example Questionnaire Items	“I worry a lot”, “I am nervous or tense”, “I am self-conscious or easily embarrassed”, “I am unhappy, sad, or depressed”, “I feel worthless or inferior”, “I cry a lot”, “There is very little that I enjoy”, “I keep from getting involved with others”	“I try to get a lot of attention”, “I talk too much”, “I damage or destroy things”, “I am impulsive or act without thinking”, “I steal”, “I lie or cheat”, “I argue a lot”, “I get in many fights”, “I am too impatient”, “I drink too much alcohol or get drunk”, “I do things that may cause me trouble with the law”
Developmental Progression	Stability of symptoms during childhood, sharp increase during adolescence and young adulthood, and a decline in older adulthood	Symptoms peak in toddlerhood, decrease during childhood, increase during adolescence, peak again during young adulthood, and steadily decline throughout mid-to-late adulthood
Broad Etiology	Heightened threat-related processing in cortico-limbic neural circuitry, blunted reward-related processing in cortico-striatal neural circuitry, and poorer prefrontal cortex executive functioning	Blunted threat-related processing in cortico-limbic neural circuitry, heightened reward-related processing in cortico-striatal neural circuitry, and poorer prefrontal cortex executive functioning

The example symptom items are taken from the Adult Self-Report questionnaire [4], which asks individuals to rate the extent to which each item was characteristic of them over the past six months on a scale of 0 (“Not True (as far as you know)”), 1 (“Somewhat or Sometimes True”), or 2 (“Very True or Often True”).

Basic science research can adopt transdiagnostic and dimensional frameworks, such as the Internalizing-Externalizing dimensions, to improve the study of the pathophysiology of psychiatric disorders. Preclinical animal models for Internalizing and Externalizing dimensions can be employed to better translate basic science findings into novel transdiagnostic targets for intervention and prevention. Unfortunately, this is not an easy feat given that the Internalizing-Externalizing framework typically is assessed using questionnaires relying on reports by the individual experiencing symptoms or by an informant (i.e., parent/guardian, teacher, clinician as in [4]). Developing objective measures of the core behavioral signs and symptoms of these dimensions across species is needed to inform animal research and is receiving increasing focus in human studies.

Transdiagnostic human research typically indicates that individuals with internalizing disorders show elevated threat-related responses, high sensitivity to punishments, and blunted responses to rewards, whereas those high in externalizing tend to show reduced responsiveness to environmental threats or punishments and heightened reward-related responses, consistent with dysfunctions in RDoC’s Negative and Positive Valence domains [1]. Thus, preclinical animal models that assess threat- and reward-related behaviors may be best able to capture the core features of the Internalizing-Externalizing framework. Examples of animal models targeting RDoC domains that map onto core signs and symptoms of Internalizing and Externalizing dimensions are outlined below.

- Internalizing
  - ◦ Animal models for anxiousness, hyperarousal, panic, and avoidance symptoms targeting threat dysfunctions within the RDoC Negative Valence System:
    - Fear conditioning (i.e., associate neutral stimulus with an aversive stimulus)
      - Expected behavior: freezing behavior and elevated heart rate to aversive stimulus (and anticipatory freezing)
    - Shock avoidance (i.e., escape latency to aversive shock)
      - Expected behavior: delayed escape latency
  - ◦ Animal models for anhedonia, dysphoria, and suicidality symptoms targeting loss within RDoC Negative Valence System and reward responsiveness within Positive Valence System:
    - Forced Swim Test
      - Expected behavior: greater immobility time floating in water vs. attempting to escape
    - Sucrose Preference Test (i.e., preference for sucrose vs. regular water)
      - Expected behavior: lack of interest in sucrose water
- Externalizing
  - ◦ Animal model of impulsivity, distractibility, and impatient urgency symptoms targeting RDoC Cognitive domain:
    - 5 choice serial reaction time task
      - Expected behavior: poor choice accuracy (selective attention) and premature responding during the waiting period (impulsive action)
  - ◦ Animal models for risk taking, excitement seeking, and substance use symptoms targeting RDoC Positive Valence System:
    - Decision-making and delay of reinforcement tasks
      - Expected behavior: tendency to seek rewards rather than avoid punishments and preference for immediate over delayed rewards
    - Novelty-preference test
      - Expected behavior: greater duration of time spent and higher level of activity in novel vs. familiar compartments
    - Conditioned place preference (i.e., conditioning of interoceptive substance cue and unique context)
      - Expected behavior: preference for context previously associated with substance following conditioning
  - ◦ Animal models for aggression and dominance symptoms targeting RDoC Systems for Social Processes:
    - Tube (i.e., competition for passage) and Water Competition tests (i.e., competition for single water source)
      - Expected behaviors: greater aggressive, dominant behaviors to “win” competition and longer time spent drinking from shared water source
  - ◦ Approach Avoidance Conflict tasks targeting both threat (Negative Valence) and reward (Positive Valence) dysfunctions may capture both Internalizing and Externalizing:
    - Elevated Plus Maze and Open Field Tests
      - Expected behavior: low approach, high avoidance (i.e., longer time spent in closed arms or “wall hugging” vs. exploration of open arms/center) in Internalizing; and high approach, low avoidance (i.e., longer time spent exploring open arms/center) in Externalizing

This list is intended to serve as a starting point for assessing the Internalizing-Externalizing dimensions in preclinical models. Ultimately, research that examines the common neurobiological mechanisms underlying a range of internalizing and externalizing symptoms and behaviors in animal models and human research [5] may be most fruitful for identifying novel transdiagnostic targets for intervention and prevention. For example, researchers can test whether neurobiological markers of animal models for depression are also markers of animal models for anxiety. This approach can help to identify the shared pathophysiology across traditional disorder categories, thereby improving clinical translation.
